# Emotional Dissonance and Sickness Absence Among Employees Working With Customers and Clients: A Moderated Mediation Model via Exhaustion and Human Resource Primacy

**DOI:** 10.3389/fpsyg.2018.00436

**Published:** 2018-04-04

**Authors:** Anne-Marthe R. Indregard, Pål Ulleberg, Stein Knardahl, Morten B. Nielsen

**Affiliations:** ^1^Department of Work Psychology and Physiology, National Institute of Occupational Health, Oslo, Norway; ^2^Department of Psychology, University of Oslo, Oslo, Norway

**Keywords:** emotion work, emotional dissonance, exhaustion, sickness absence, organizational climate

## Abstract

Emotional dissonance, i.e., a discrepancy between required and felt emotions, has been established as a predictor of sickness absence in studies, but little is known about mechanisms that can explain this association. In order to prevent and reduce the impact of emotional dissonance on sickness absence, there is a need for greater attention to variables explaining when and how emotional dissonance is related to sickness absence. The overarching aim of this study was to examine whether emotional dissonance has an indirect association with sickness absence through exhaustion. In addition, we examined whether human resource primacy (HRP), which is the employer’s degree of concern for human resources, moderates this indirect effect. A sample of 7758 employees, all working with customers and clients, were recruited from 96 Norwegian organizations. Emotional dissonance, exhaustion, and HRP were measured through surveys and then linked to registry data on medically certified sickness absence for the year following the survey assessment. Results showed that exhaustion is a mediator for the relationship between emotional dissonance and sickness absence. Furthermore, higher levels of HRP were found to reduce the positive association between emotional dissonance and exhaustion, and the indirect effect of emotional dissonance on sickness absence through exhaustion is found to be weaker when HRP is high. By testing this moderated mediation model, the current study contributes to the literature on emotion work by clarifying mechanisms that are crucial for the development of targeted interventions that aim to reduce and prevent sickness absence in client-driven work environments.

## Introduction

Since the early 1970s, there has been a major redistribution of employment from industry into the service sector (Dolphin, 2015), and service jobs have become the major form of employment in European countries (Paoli and Merllié, 2012). Through their direct contact with customers and clients, service employees are expected to display emotions according to the organization’s explicit or implicit emotional display rules (Brotheridge and Grandey, 2002; Ortiz-Bonnín et al., 2016). Regulating feelings to display the appropriate emotion during social interactions has thereby emerged as a job demand for many employees (Zapf, 2002). Emotion regulation as part of the work role has been referred to as emotion work (emotional labor; Hochschild, 1983). Emotion work is a multifaceted construct which may have positive as well as negative consequences for both employees and organizations (Zapf and Holz, 2006). With regard to health outcomes, the key dimension of emotion work is emotional dissonance (Hochschild, 1983; Zapf, 2002), and is defined as the discrepancy between required and felt emotions (Morris and Feldman, 1996; Zapf and Holz, 2006). Accumulated evidence suggests that regulation of feelings at the workplace increases the risk of feeling exhausted (Zapf, 2002; Hülsheger and Schewe, 2011) and being absent from work (Nguyen et al., 2013; Indregard et al., 2016).

Absenteeism refers to the failure to report to work as scheduled (Johns, 2010). Sickness absence is a major contributor to reduced workplace productivity and has considerable costs for individuals and society (Darr and Johns, 2008). Given the serious consequences of absenteeism, substantial research effort has been dedicated to better understand and predict its occurrences. Emotional dissonance is a potential antecedent as studies have demonstrated a direct relationship between emotional dissonance and sickness absence (Indregard et al., 2016). However, in order to prevent and reduce sickness absence, there is need for greater attention to conditions and mechanisms explaining when and how emotional dissonance is related to sickness absence. Furthermore, it is important to identify factors that are possible to modify for the organization.

**FIGURE 1:**
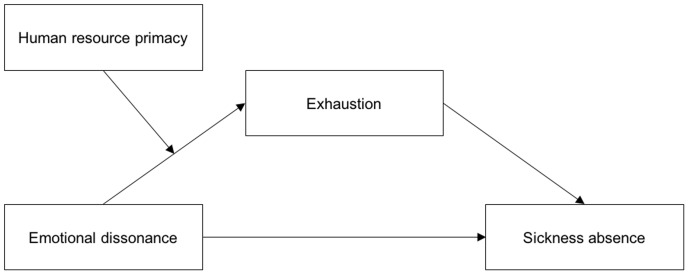
A graphical conceptual overview of the moderated mediation model.

The overarching aim of the study was to gain more insight into the relationship between emotional dissonance and sickness absence. Building on Conservation of Resources (COR) theory (Hobfoll, 1989), we will examine exhaustion as a mediating factor in the relationship between emotional dissonance and sickness absence. In addition, we will examine whether human resource primacy (HRP) moderates the direct relationship between emotional dissonance and exhaustion, as well as the indirect relationship between emotional dissonance and sickness absence. HRP refers to the employer’s degree of concern for human resources (Flamholtz, 1999). By examining these associations, this study will extend previous research by showing *how* and *under which conditions* emotional dissonance is related to sickness absence. Much of the previous research on emotion work is based on same-source data, potentially subjected to common method bias (Podsakoff et al., 2003). Using a combination of questionnaire survey- and objective registry data on sickness absence, the present study will be a methodological contribution to the literature on emotion work. A graphical overview of the described associations is included in **Figure 1**. In the following, we will elaborate on the relationships in more detail and present our study hypotheses.

### The Mediating Role of Exhaustion in the Relationship Between Emotional Dissonance and Sickness Absence

A relationship between emotional dissonance and sickness absence has previously been reported, showing that higher levels of emotional dissonance are related to an increased risk of sickness absence among employees working with customers and clients (Indregard et al., 2016). In a study of employees working in financial services, Diestel and Schmidt (2010) found an interactive effect of emotional dissonance and self-control demands on sickness absence. In a study among hospital nurses, Nguyen et al. (2013) found surface acting (i.e., a strategy used when the employee does not attempt to modify feelings to match the required displays) to be directly related to increased sickness absence. Even though a relationship between emotional dissonance and sickness absence is found, there is a shortage of existing research on the mechanisms that can explain how emotional dissonance can lead to sickness absence. However, there are both theoretical and empirical reasons for considering emotional exhaustion as a key intervening variable in the relationship.

Research has demonstrated that employees engaged in emotion work are at higher risk of experiencing occupational burnout (Zapf, 2002). Burnout has been defined as “a state of physical, emotional, and mental exhaustion that results from long-term involvement in work situations that are emotionally demanding” (Pines and Aronson, 1988). According to Maslach (1982), burnout is a syndrome consisting of the dimensions emotional exhaustion, depersonalization or cynicism (felt distance from others), and reduced accomplishment. Exhaustion is considered the core element of the burnout (Pines and Aronson, 1988; Schaufeli and Greenglass, 2001; Kristensen et al., 2005), and it represents a state of physical and emotional depletion resulting from long-term exposure to excessive job demands and continuous hassles (Lee and Ashforth, 1996; Zohar, 1997). Theoretically, the association between emotional dissonance and exhaustion can be explained with COR theory (Hobfoll, 1989; Hobfoll et al., 2018). The central tenet of COR theory is the view that individuals strive to obtain, retain, and protect valued resources, including emotional resources, and minimize the risk of resource loss (Hobfoll, 2004). When employees work under demanding situations caused by interpersonal interactions that require frequent emotion regulation, a discrepancy between experienced and expressed emotions may lead to low energy and mental fatigue (Chi and Liang, 2013). According to COR theory, exhaustion occurs through prolonged periods of having few resources, which leads other resources to become compromised as well (Arnold et al., 2015). Consequently, the experienced exhaustion may further deplete the necessary resources the employee needs to cope with emotional demands in the workplace. Taken together, the employee may experience emotional exhaustion because of an imbalance between emotional demands in the form of emotion regulation and the resources available to cope with such demands.

Regulating emotions may be an effortful process that drains mental resources and thereby enhances strain (Grandey, 2003; Coté, 2005). When valued resources are threatened or lost as a result of emotion regulation, employees will actively strive to prevent further resource loss by protecting themselves from the resource depleting work situation (Hobfoll, 1989). Being away from work can be a way of preventing further resource loss and might also be restorative by providing the chance to reload depleted resources (Nguyen et al., 2013). Thus, employees feeling exhausted due to frequently experiencing emotional dissonance may have a higher risk of sickness absence.

Empirically, emotional dissonance has been firmly established as a correlate of exhaustion in previous research (see the literature review by Zapf, 2002 and meta-analysis by Hülsheger and Schewe, 2011). Although it is generally assumed that exhaustion is related to health complaints and increased risk of sickness absence, this link remains somehow less studied than the relationship between emotion work and exhaustion. However, existing studies seem to provide relatively consistent support for positive association between exhaustion and sickness absence (Schaufeli and Enzmann, 1998; Ahola et al., 2008; Glise et al., 2010; Roelen et al., 2015).

Based on the above theoretical considerations and review of existing empirical evidence, we hypothesized that emotional dissonance is a predictor of sickness absence, through the experience of exhaustion. Thus, we expect that employees exposed to emotional dissonance experience increased levels of exhaustion which subsequently leads to increased risk of sickness absence. Hence:

     *H1: Emotional dissonance has a positive indirect effect on sickness absence through emotional exhaustion.*

### The Moderating Role of Human Resource Primacy

Although it is reasonable to assume that emotional dissonance leads to exhaustion and sickness absence, it is unlikely that all employees will react to emotional dissonance in the same manner. Rather, following well-established stress process models, such as the Transactional Model of Stress and Coping (Lazarus and Folkman, 1984), the effects of emotional dissonance on health will be dependent on moderating variables, including individual and organizational characteristics. Emerging research suggest that organizational climate may be especially important with regard to employee health and well-being (Schneider et al., 2013). Organizational climate is a multidimensional construct including a wide range of individual experiences and evaluations of the work environment (James and James, 1989). The employee’s evaluations may refer to different facets of the work environment, such as perceived organizational support or leadership. HRP represents a specific facet of organizational climate that may be especially important with regard to employee well-being (Bedeian et al., 1981; Birkeland et al., 2017; Nielsen and Knardahl, 2017). Conceptually, HRP is a form of organizational support that refers to whether an employer communicates interest in their employees’ health, well-being, and happiness (Kline and Boyd, 1991). If HRP is high, there is a central concern for human resources. In contrast, if HRP is low, human resources are of secondary importance (Flamholtz, 1999).

According to COR theory, employees use their personal resources to cope with stressful situations and exert control over their work environment in order to obtain new resources to fulfill their valued needs (Hobfoll, 1989; Hobfoll and Shirom, 2001). When valued personal resources are depleted, HRP can serve as an additional coping resource that can help employees manage the emotional demands. In a theoretical model of the role of emotional dissonance in organizational behavior, Abraham (1998) proposed that employees that experience high social support are less likely to be negatively affected by emotional dissonance. This assumption, that a positive organizational climate will buffer the impact of emotional demands on employee health, is further supported by some research findings. Hochschild (1983) proposed that an environment where the employees perceived strong social support would enable the employees to vent frustrations about customers and clients. Emotional support provided by the environment helped to lessen the strain associated with having to regulate one’s feeling when interacting with an obnoxious customer or client. Kinman et al. (2011) showed that social support buffers the negative impact of emotional demands on emotional exhaustion among teachers. Ortiz-Bonnín et al. (2016) recently found in their study of hotel employees that a supportive climate had a buffering effect on the relationship between emotional dissonance and emotional exhaustion, meaning that supportive climate protects employees who experience emotional dissonance from suffering emotional exhaustion.

On this empirical and theoretical basis, we propose that when employees perceive that the organization is interested in their health and well-being, i.e., that their organization has high HRP, the negative effects of emotional dissonance on emotional exhaustion will be attenuated. Consequently, we hypothesized that

     *H2: Human resource primacy moderates the relationship between emotional dissonance and exhaustion, i.e., the relationship between emotional dissonance and exhaustion is weaker under conditions of high levels of human resource primacy*.

According to COR theory, employees may enter a defensive mode if their resources are overstretched or exhausted (Hobfoll et al., 2018). When the employee experiences high resource loss, resource gain becomes more important. When an employee feels exhausted, sickness absence may become a survival strategy to withdraw from a stressor. According to COR theory, employees with greater resources available are less vulnerable to resource loss and more likely to gain resources (Hobfoll et al., 2018). Thus, having to withdraw from the resource depleting work situation may be less necessary. HRP works by promoting predictability and perceived control, as a strong HRP indicates that employees perceive their management to be motivated and obliged to intervene in difficult working situations and that employees perceive the conflict management procedures to be fair (Einarsen et al., 2016). In work environments where the employee experiences a strong HRP, employees will know that they can seek support and advice from the management in difficult situations rather than relying on their own coping strategies. Consequently, perceiving the HRP as strong provides an experience of control that should attenuate the indirect effect of emotional dissonance on sickness absence through emotional exhaustion by reducing the level of experienced strain in the situation. Therefore, we hypothesized that

     *H3: The indirect effect of emotional dissonance on sickness absence through exhaustion is moderated by human resource primacy, i.e., the relationship is weaker under conditions of high levels of human resource primacy.*

## Materials and Methods

### Design and Study Sample

The current study is a part of the research project: “The new workplace: Work, health, and participation in the new work life,” a longitudinal web-based survey carried out by the National Institute of Occupational Health (see Christensen and Knardahl, 2010; Finne et al., 2014; Emberland and Knardahl, 2015). The study design for the present study was prospective, with all psychological and social work factors measured at baseline, and then linked to official registry data on sickness absence for the year following the survey assessment. For a more detailed description of the research project, see study protocol published elsewhere (Nielsen et al., 2016). Organizations were contacted by the National Institute of Occupational Health and offered to participate in the study. After information about the general study aims was given at the organizational level, each employee, excluding those on sick leave, received a letter containing information about the survey, the strict confidentiality guidelines, as well as information about the license for data collection granted by the Norwegian Data Inspectorate. A written consent was obtained before linking survey questionnaire to registry data on sickness absence. A detailed description of the recruitment has been published elsewhere (Christensen and Knardahl, 2010).

A total of 30,945 adult employees in a full time or part time position, from 96 organizations, were invited to participate in the survey. Altogether 15,302 persons responded (response rate: 49.4%). In the present study, only employees who reported working with customers and clients and who answered the items measuring emotional dissonance were included (*n* = 10,781). Of these, 7,758 (71.6%) respondents permitted linking survey data to registry data on sickness absence. About 85% of the sample responded to the survey using the electronic survey form. A paper version of the questionnaire was sent out if requested in advance.

The study sample consisted of more women (59.7%) than men (40.3%), and the mean age was 42.7 (*SD* = 10.59). About 52% had minimum 13 years of education, 82.4% were permanently employed, and the majority did not have management responsibilities (82.6%). About 84.2% had direct contact (face-to-face) with customers and clients while 15.8% had mostly indirect contact (phone, e-mail). Of all employees, 39.9% had at least one day with medically certified sickness absence within the year following the survey measurement.

### Measures

#### Sickness Absence

Information on medically certified sickness absence was accessed through the Norwegian Labor and Welfare Administration (NAV). The registry provides complete registrations of all medically certified sickness absence from the first day absent, including the length and medical diagnosis. The registry should be accurate since correct registration is required for the transfer of payments by the social insurance scheme. We aggregated data on sickness absence over a 12-month follow-up post-survey, which is consistent with previous research (Diestel and Schmidt, 2010; Nguyen et al., 2013). Registry information of sickness absence was linked to the survey data by the unique 11-digit national individual identity number. The time period the employees were eligible for sickness absence was considered the same for all respondents within each company, starting from the day the electronic forms were closed. The registry was checked for inconsistencies. Overlapping or duplicate spells of sickness absence were merged.

#### Emotional Dissonance

Emotional dissonance was measured by five items (α = 0.89) adapted from the Frankfurt Emotion Work Scales (Zapf et al., 1999), example item: “How often in your job do you have to suppress emotions in order to appear neutral on the outside?.” Responses were provided on a 5-point scale with the following alternatives “1 = seldom or never,” “2 = once per week,” “3 = once per day,” “4 = several times per day,” and “5 = several times an hour.” Evidence for criterion-related validation of the scale has been showed by Zapf et al. (1999).

#### Exhaustion

A sub-dimension from the Copenhagen Burnout Inventory (CBI) (Kristensen et al., 2005) was used to measure exhaustion, example items: “How often do you feel tired?” and “How often do you feel worn out?”. The dimension, personal burnout, consists of six questions measuring fatigue and exhaustion. Cronbach’s α was 0.84. Personal burnout is regarded as a state of prolonged physical and psychological exhaustion (Winwood and Winefield, 2004). The measurement does not attempt to distinguish between physical and psychological exhaustion and the experience of exhaustion is not a phenomenon restricted to human service professions (Kristensen et al., 2005). Answer categories ranged from 1 to 5, where 1 = very seldom or never, and 5 = nearly every day.

#### Human Resource Primacy

The HRP was measured with an established 3-item scale from the General Nordic Questionnaire for Psychological and Social Factors at Work (QPSNordic). Cronbach’s α was 0.77. The concept was measured by the following three items: (1) “At your organization, are you rewarded (money, encouragement) for a job well done?”; (2) “Are workers well taken care of in your organization?”; and (3) “To what extent is the management of your organization interested in the health and well-being of the personnel?” (Dallner et al., 2000). Responses were recorded with the following alternatives: 1 = “very little or not at all,” 2 = “rather little,” 3 = “somewhat,” 4 = “rather much,” and 5 = “very much”.

#### Covariates

Covariates included in the multivariable models were selected on the basis of past research (Allebeck and Mastekaasa, 2004; Duijts et al., 2007). The variables included were gender, age (measured continuously in years), and sickness absence history (days absent the year before questionnaire measurement).

### Statistics

Study hypotheses were tested using Mplus Version 7.4 (Muthén and Muthén, 2015). First, we examined a mediation model with exhaustion as mediator in the relationship between emotional dissonance and sickness absence (*H1*). Furthermore, we tested whether HRP moderated the association between emotional dissonance and exhaustion (*H2*). Finally, a moderated mediation model was tested (*H3*). Our dependent variable, the number of days absent the year after answering the questionnaire, is a discrete count variable. Poisson distributions are commonly used to analyze count outcome, including sickness absence data (Marmot et al., 1995; North et al., 1996; Kivimaki et al., 2001; Rugulies et al., 2007). However, Poisson regressions require that the variance is equal to the mean. For sickness absence data, the variance is frequently substantially larger than the mean, a condition known as overdispersion (Cameron and Trivedi, 1998). Second, the variable includes more values of zeros than expected from the Poisson distribution. To overcome both overdispersion and excess zero-values, a Zero-Inflated Negative Binomial (ZINB) was used both in the mediation model and in the moderated mediation model.

The use of a dependent variable defined as a zero-inflated count variable makes the estimation of indirect effect and moderated mediation effect more complicated than conventional mediation and moderated mediation analysis, see Preacher et al. (2007) for a description of conventional analysis. Therefore, the approach described by Muthén et al. (2017) was applied. Using the MODEL INDIRECT command in Mplus, we estimated causally defined direct, indirect, and total effects, which should be used when the dependent variable is defined as a count variable.

The subjects were clustered in organizations and are likely to have similar characteristics. Thus, lack of independence between observations may exist in the data. Taking into account non-independents of observations due to cluster sampling, we computed cluster-adjusted standard errors using a sandwich estimator (Asparouhov and Muthen, 2006). Because we examine the employees’ perception of the organization’s interest in their health and well-being and how this is related to sickness absence, organization affiliation was used as the cluster variable. This clustering was taken into account in all analyses.

## Results

### Descriptive Statistics

The means, standard deviations (SD), and intercorrelations between the variables included in the moderated mediation model are listed in **Table 1**. Emotional dissonance showed positive relationship with exhaustion (*r* = 0.23; *p* < 0.01) and negative relationship with HRP (*r* = -0.24; *p* < 0.01) and age (*r* = -0.13; *p* =< 0.01). Exhaustion showed positive relationships with sickness absence (*r* = 0.17; *p* < 0.01), sickness absence history (*r* = 0.19 *p* =< 0.01), and sex (*r* = 0.10; *p* =< 0.01), and negative relationship with HRP (*r* = -0.25; *p* < 0.01). Remaining intercorrelation were considered as small and/or insignificant.

**Table 1:** Means (M), standard deviations (SD), and intercorrelations for variables included in the moderated mediation model.

Variables	Descriptive		Correlations					
	*M*	*SD*	1	2	3	4	5	6
1 Emotional dissonance	2.53	0.90						
2 Exhaustion	1.85	0.70	0.23**					
3 Sickness absence (days)	24.67	66.51	0.05**	0.17**				
4 Human resource primacy	3.07	0.90	-0.24**	-0.25**	-0.06**			
5 Sickness absence history (days)	17.08	50.76	0.05**	0.19**	0.10**	-0.05**		
6 Age	42.69	10.59	-0.13**	-0.03**	0.04**	0.04**	0.05**	
7 Sex (1 = male 2 = female)			0.02*	0.10**	0.09**	0.05**	0.09**	-0.05**

*N = 7339; ^∗∗^p < 0.01.*

### The Mediation Effect of Exhaustion on the Relationship Between Emotional Dissonance and Sickness Absence

Results from the mediation analysis are presented in **Table 2**. A significant indirect effect of emotional dissonance on sickness absence through exhaustion was established; *b* = 0.38; 95% CI: 0.23, 0.53; *p* < 0.001 (*H1*). This meant that an increase in emotional dissonance was related to an elevation in exhaustion, which in turn was expected to result in an increase in sickness absence. The direct effect of emotional dissonance on sickness absence (*b* = -0.098) and the total effect (*b* = 0.29) were not statistically significant in the tested mediation model. Since the dependent variable (sickness absence) was defined as a zero-inflated count variable, the estimation of the indirect effect has to be done differently than conventional mediation analysis. The approach described by Muthén et al. (2017) was applied, estimating causally defined indirect, direct, and total effects.

**Table 2:** Mediation of the association between emotional dissonance (ED) and sickness absence (SA) through exhaustion (EX).

Effects of ED on SA		Estimate	95% CI	*p*-value
Indirect effect	ED→EX→SA	-0.38	-0.23, 0.53	0.000
Direct effect	ED→SA	-0.098	-0.51, 0.32	0.699
Total effect	Indirect + direct effect	-0.29	-0.12, 0.69	0.247

*N = 7339; Unstandardized estimates.*

### The Moderating Effect of Human Resource Primacy on the Relationship Between Emotional Dissonance and Exhaustion

**FIGURE 2:**
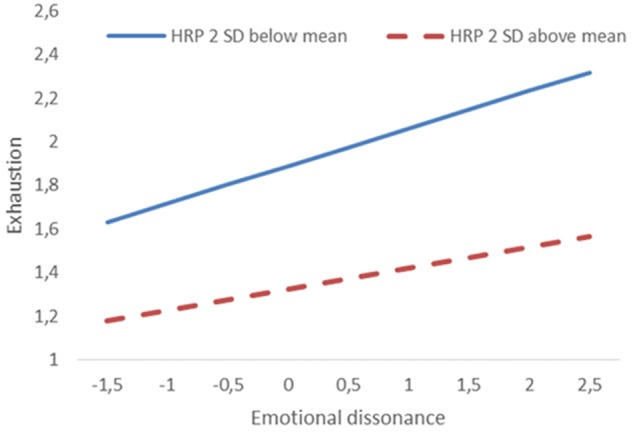
Moderator effect of human resource primacy (HRP) on the relationship between emotional dissonance and exhaustion. HRP and emotional dissonance are mean centered. Both slopes are significant (*p* < 0.001).

The relationship between emotional dissonance and exhaustion, and whether the level of HRP would moderate this relationship (*H2*), was estimated using a multiple regression model. A significant association between emotional dissonance and exhaustion was first established; *b* = 0.17; 95% CI: 0.16, 019; *p* < 0.001. HRP was found to significantly moderate the relationship between emotional dissonance and exhaustion; *b* = -0.021; 95% CI: -0.038, -0.004; *p* < 0.001. The negative coefficient demonstrated that higher levels of HRP reduced the positive association between emotional dissonance and exhaustion, while the association was stronger at lower levels of HRP, as illustrated in **Figure 2**.

The moderating effect of HRP was tested by including an interaction term between emotional dissonance and HRP. Both variables were mean centered to aid the interpretability of the results. The effects of sex, age, and sickness absence history were controlled for in the moderation model.

### Tests of Moderated Mediation Model

Finally, we combined the mediation model and the moderation model to test whether the strength of the indirect effect, from experiencing emotional dissonance to sickness absence through exhaustion, could be conditional on values of HRP (*H3*). All path analyses in the full moderated mediation model are presented in **Table 3**. Conventionally, a moderated mediation index can be calculated by multiplying the path from predictor to mediator with the path from mediator to outcome, but since outcome (sickness absence) is defined as a zero-inflated count variable, the estimation method descried by Muthén et al. (2017) was applied. A statistically significant effect of emotional dissonance on exhaustion (*b* = 0.134, *p* < 0.001) was established, and emotional dissonance was significantly related to sickness absence (*b* = 0.241, *p* < 0.001). The pseudo-*R*^2^ of the model was 0.267, representing the percentage reduction in deviance for the model with all predictors compared to a model without predictors (intercept only model).

**Table 3:** Regression coefficients and incidence rate ratio with confidence intervals for paths included in the full moderated mediation model.

	Exhaustion		Sickness absence			
	*b* (unstandardized reg. coeff.)	95% CI	*b* (unstandardized reg. coeff.)	95% CI	Incident rate ratio (IRR)	95% CI
Emotional dissonance	-0.134***	-0.110, -0.157	-0.017	-0.079, 0.044	0.983	0.922, 1.043
Human resource primacy	-0.157***	-0.181, -0.133	-0.030	-0.096, 0.037	0.971	0.906, 1.035
Emotional dissonance × human resource primacy	-0.021*	-0.038, -0.004				
Exhaustion			0.241***	0.137, 0.345	1.273	1.140, 1.405
**Control variables**						
Age	0.000	-0.002, 0.003	0.013***	0.010, 0.017	1.013	1.010, 1.017
Gender (1 = male, 2 = female)	0.131***	0.087, 0.175	0.201**	0.073, 0.330	1.223	1.066, 1.380
Absence history	0.002***	0.002, 0.003	0.002***	0.001, 0.003	1.002	1.001, 1.003

*N = 7339. Emotional dissonance and human resource primacy are mean centered. ^∗^p < 0.05; ^∗∗^p < 0.01; ^∗∗∗^p < 0.001.*

The estimated indirect effects (from emotional dissonance to sickness absence through exhaustion) at different levels of HRP (moderator variable) are presented in **Table 4**. The results show that the indirect effect from emotional dissonance to sickness absence through exhaustion decreases (from *b* = 0.41; 95% CI: 0.16, 0.65; *p* = 0.002 to *b* = 0.17; 95% CI: 0.07, 0.28; *p* = 0.001) with higher levels of HRP. Still, the indirect effect is significant at all levels of HRP. All effects are adjusted for sex, age, and sickness absence history.

**Table 4:** Moderated mediation effect of emotional dissonance on sickness absence at specific conditional values of human resource primacy.

Specific conditional values of human resource primacy	Indirect effect	95% CI
-2 SD below the mean	0.405^∗∗^	0.161, 0.649
-1 SD below the mean	0.338^∗∗^	0.142, 0.533
Mean	0.277^∗∗∗^	0.122, 0.433
+1 SD above the mean	0.223^∗∗^	0.098, 0.348
+2 SD above the mean	0.174^∗∗^	0.070, 0.278

*Causally defined indirect effects. N = 7339. Emotional dissonance and human resource primacy are mean centered. ^∗∗^p < 0.01; ^∗∗∗^p < 0.001.*

## Discussion

The overarching aim of the present study was to gain more insight in mechanisms that can explain the relationship between experiencing emotional dissonance at work and sickness absence. To our knowledge, few studies have investigated mechanisms pertaining to explain the direct relationship between emotional dissonance and sickness absence. As an extension of previous research, and building on the COR theory (Hobfoll, 1989), the current study examined exhaustion as a mediating factor in the relationship between emotional dissonance and sickness absence. Furthermore, HRP was considered an additional resource and tested as a moderator of the direct relationship between emotional dissonance and exhaustion, as well as a moderator of the indirect relationship between emotional dissonance and sickness absence through exhaustion. By testing this moderated mediation model, the current study contributes to the literature on emotion work by clarifying mechanisms that are crucial for the development of targeted interventions aiming to reduce and prevent sickness absence in client-driven work environments.

Consistent with our first hypothesis, the results show that experiencing emotional dissonance at work is related to medically certified sickness absence through the experience of exhaustion. Hence, the present study, using a combination of survey data and objective registry data on sickness absence, links previous research that has demonstrated direct relationships between emotional dissonance and exhaustion (Zapf, 2002; Hülsheger and Schewe, 2011) to research on the relationship between exhaustion and sickness absence (Kristensen et al., 2005; Ahola et al., 2008; Glise et al., 2010). Having to express emotions that are not genuinely felt requires psychological effort and arousal (Gross, 1998) which may deplete the employees physical and emotional resources (Zapf and Holz, 2006). Grandey (2000) suggested that over time, these depleting effects from performing emotion work may lead to sickness absence. Absenteeism is a way to escape from or compensate for aversive work circumstances (Bakker and Heuven, 2006). In line with Nguyen et al. (2013), this study shows that being away from work may be a way to prevent further resource loss, as well as a restorative strategy by giving the opportunity to reload depleted resources. In the framework of COR theory (Hobfoll, 1989; Hobfoll and Freedy, 1993), emotional dissonance is considered detrimental because it entails emotion regulation, an effortful process that drains emotional and physiological resources. Employees have to invest resources to meet emotional demands arising from client interactions. The amount of resources spent with customers and clients represent a loss of existing resources. Employees will use available resources to obtain control over their work situation and to gain new resources. In situations where the employee does not have resources available to gain new ones, absence from work is a strategy to prevent further resource loss. According to Hobfoll’s perspective (Hobfoll and Shirom, 2001), absenteeism is considered a protective mechanism when valued resources are threatened or lost.

Our second hypothesis was confirmed when we found a moderating effect of HRP on the well-established relationship between emotional dissonance and exhaustion. More precisely, the results show that higher levels of HRP reduce the positive association between emotional dissonance and exhaustion, while the association is stronger at lower levels of HRP. Finally, in support of our third hypothesis, the test of the moderated mediation model showed that the indirect effect of emotional dissonance on sickness absence through exhaustion depends on values of HRP.

The finding that high levels of HRP act protective on the relationship between emotional dissonance and exhaustion, may be explained by HRP’s contribution to a supportive work environment. Being part of a positive and supportive work environment, employees probably have more resources available when interacting with customer and clients. Hochschild (1983) proposed that a work environment where the employees perceived strong social support would enable the employees to vent frustrations. Kinman et al. (2011) have for instance showed that provision of emotional monitoring increases in a supportive environment.

Cheung et al. (2011) showed in their study that COR is necessary for an employee to cope with emotional demands at work. A core assumption in COR theory (Hobfoll, 1989) is that those employees who possess more resources have better health outcomes and are, in general, better off than those with fewer resources. Individuals’ well-being and work behaviors are dependent on their perceived access to resources. In this context, HRP reflects a work environment with more resources available that buffer the negative impact of emotional dissonance. Hence, in the view of COR theory, our findings show that HRP can represent a form of additional and secondary resource that can help employees manage emotional demands in situations when the personal resource pool is drained. Specifically, our results show that high levels of HRP seem to be beneficial with regard to reducing the risk of exhaustion and sickness absence among those employees experiencing frequent emotional dissonance.

### Limitations and Suggestions for Future Studies

There are some strengths and limitations that should be addressed in the interpretation of the findings. First, the study variables that measured the predictor, the mediator, and the moderator relied on survey-based self-report data and were measured from the same source. As such, common method variance may inflate the relations between constructs (Podsakoff et al., 2003). The fact that sickness absence was measured by objective registry data is therefore a significant strength of the study, since the combination of survey data and objective registry data reduces the risk of observing spurious associations (Podsakoff et al., 2003).

Second, emotional dissonance and exhaustion were measured at the same time point and the mediation analysis is consequently limited in their ability to test causal mechanisms (Little, 2013). Although prior studies provide support for the direction from emotional dissonance to exhaustion (Zapf, 2002; Hülsheger and Schewe, 2011), future longitudinal studies would provide stronger evidence for the directionality of the associations found in our study.

Third, our hypotheses examined only one pathway connecting emotional dissonance to sickness absence, whereas several others could be in play as well. Although the results from our study are consistent with the hypothesized relationships in the model, there may be alternative explanations for the relationships in the model. For instance, due to individual dispositions it is unlikely that all employees react to emotional dissonance in the same manner. Prior studies have demonstrated that the resource depleting effects of emotion regulation processes are not equal for all employees (Nguyen et al., 2013). Future studies should therefore address individual differences that may influence the strength of the relationship between emotional dissonance and health outcomes.

Finally, the magnitude of the detected indirect association between experiencing emotional dissonance at work and sickness absence was small. However, because the causes of sickness absence are multifactorial and many variables will therefore be accounting for sickness absence, it is unreasonable to expect that one single variable should explain the “lion’s share” of variation in sickness absence (Zapf et al., 1996; Beehr, 2014). Although only a small part of the variance in sickness absence is explained by emotional dissonance, it is important to note that the impact of having to regulate one’s emotions at work may still be substantial and has important practical relevance (Cortina and Landis, 2009).

## Implications

Despite the limitations listed above, the findings from the current study have important methodological, theoretical, and practical implications. Analyzing factors related to sickness absence using structural equation modeling (SEM) allows for testing of more complex models as compared to direct effect models or models that examine moderation and mediation separately. Together with the theoretical framework provided by COR theory, the study tests hypotheses that contribute to the knowledge on intermediate factors explaining the process from experiencing emotional dissonance to sickness absence. By also including a moderator, the tested model gives information about a modifiable work factor that can buffer this process. This is especially relevant knowledge with regard to reducing and preventing exhaustion and sickness absence among employees working directly with customers and clients. The service sector is growing and an increasing proportion of employees work directly with people. Therefore, it is important to identify the specific work factors that are central in service occupations. Further, to ensure health-promoting work places it is crucial to know how and when work factors affect employee health and well-being. According to the findings from the present study, organizations should be encouraged to invest in their employees’ health and well-being by developing an organization that communicates interest in employees’ resources. Such an investment could reduce the negative effect of emotional dissonance on health outcomes and further reduce sickness absence.

## Conclusion

The current study revealed that exhaustion is a mediator for the relationship between emotional dissonance and sickness absence, and more importantly, that this indirect relationship is dependent on HRP. By testing this moderated mediation model, the current study contributes to the literature on emotion work by clarifying mechanisms that are crucial for the development of targeted interventions aiming to reduce and prevent sickness absence in client-driven work environments.

## Ethics STATEMENT

This project has been approved by the Regional Committees for Medical and Health Research Ethics (REC) in Norway (REC South East), has permission from The Norwegian Data Protection Authority, and was conducted in with the World Medical Association Declaration of Helsinki. All study participants provided their informed consent.

## Author Contributions

A-MI contributed to the design of the study, performed the analysis and interpretation of the data, and wrote the first draft of the manuscript. SK contributed to the design of the study, contributed to the data collection, and critically revised the manuscript. MN contributed to the design of the study, contributed to the statistical analysis, and critically revised the manuscript. PU and MN contributed to the design of the study, contributed to the statistical analysis, and critically revised the manuscript.

## Conflict of Interest Statement

The authors declare that the research was conducted in the absence of any commercial or financial relationships that could be construed as a potential conflict of interest.
